# Individual and situational predictors of psychological and physiological stress and burnout among maternity providers in Northern Ghana

**DOI:** 10.1371/journal.pone.0278457

**Published:** 2022-12-15

**Authors:** Jerry John Nutor, Raymond A. Aborigo, Jaffer Okiring, Irene Kuwolamo, John Baptist K. Dorzie, Monica Getahun, Wendy Berry Mendes, Patience A. Afulani

**Affiliations:** 1 Department of Family Health Care Nursing, University of California, San Francisco, San Francisco, California, United States of America; 2 Navrongo Health Research Centre, Navrongo, Ghana; 3 Infectious Diseases Research Collaboration, Kampala, Uganda; 4 Institute for Global Health Sciences, University of California, San Francisco, San Francisco, California, United States of America; 5 Department of Psychiatry and Behavioral Sciences, University of California, San Francisco, San Francisco, California, United States of America; 6 Department of Epidemiology & Biostatistics, University of California, San Francisco, San Francisco, California, United States of America; 7 Department of Obstetrics, Gynecology & Reproductive Sciences, University of California, San Francisco, San Francisco, California, United States of America; Public Library of Science, UNITED STATES

## Abstract

**Background:**

Maternity providers, including nurses, midwives, physicians, are at significant risk for stress and burnout due to the nature of care provision in maternal and child health settings. Yet, the empirical evidence on stress and burnout among maternity providers in sub-Saharan Africa is scarce. Therefore, the purpose of our study was to (1) assess levels of stress and burnout among maternity providers and support staff in Ghana, and (2) identify individual and situational factors associated with maternity provider stress, burnout, and physiology.

**Method:**

Using a purposive sampling technique, we recruited 150 maternity providers from 19 high delivery health facilities within the 15 districts of the Upper East region (UER) of Ghana into a cross-sectional study. Participants completed Cohen’s Perceived Stress Scale, the Shirom-Melamed Burnout scale, and sociodemographic, health-, and work-related items. Participants’ heart rate variability (HRV) and hair cortisol levels were assessed for stress-related physiologic responses. We computed bivariate and multivariate linear regression models to examine factors associated with stress and burnout.

**Result:**

Most participants were experiencing moderate to high stress (58.0%) and burnout (65.8%). Each unit increase in overcommitment to work was associated with 0.62 higher perceived stress scores (β = 0.62, 95% CI: 0.22, 1.02) and 0.15 higher burnout scores. On average, those who had experienced disrespect from colleagues in the last year had higher perceived stress scores compared to those who had not experienced disrespect (β = 1.77, 95% CI: 0.50, 3.04); and those who had experienced disrespect from patients in the last year had higher cortisol levels than those who had not (β = 0.52, 95% CI: 0.11, 0.93). Those who work for more than 5 days also had higher cortisol levels, on average, compared to those who worked fewer days a week.

**Conclusion:**

We found high levels of stress and burnout among maternity providers in Northern Ghana, underscoring the need for interventions to manage the effects of stress and burnout on maternity providers’ wellbeing, quality of care, and patient outcomes. Healthcare management teams should assist providers in reducing their overcommitment by hiring more staff, given its strong link to stress and burnout. Additionally, facilitating a respectful workplace culture could also help reduce stress and burnout among maternity providers.

## Introduction

Stress, the state of psychological and physiological strain or tension resulting from adverse or demanding circumstances [1], impacts healthcare providers globally. Scientific evidence on the physiological responses to stress has demonstrated the role of the hypothalamic-pituitary-adrenal (HPA) axis and the autonomic nervous systems (ANS) in the stress response [2, 3]. The effects of stress is further mediated by how people perceive stress and their physiological responses [4, 5]. Research has also shown the impact of prolonged stress, including physical and psychological effects, deterioration in emotional functioning, and in the case of healthcare providers, difficulty in adapting to professional settings and subsequent burnout [6].

The persistent stress of healthcare provision predisposes providers to burnout, which manifests as physical, emotional, and mental exhaustion [7]. Further, stress and burnout has been shown to lead to anxiety [8], depression [9, 10], substance abuse and suicidal ideation [10, 11], gastrointestinal disorders [12, 13], cardiovascular diseases [13], poor quality of life, and premature death [13, 14]. Burnout among providers manifests in various ways including (1) minimizing one’s impact (viewing care as either negative or ineffective [15]); (2) emotional exhaustion, including the lack of energy and enthusiasm [16, 17]; and (3) detachment from role, cynicism, and depersonalization [15]. Clinically, burnout among providers has been linked to negative health outcomes for both patients and providers, frequently attributed to decreased effort and time towards care provision [7, 18]. Further, burnout has profound impacts on healthcare systems leading to high rates of absenteeism, turnover, and increased costs [16, 19, 20]. These outcomes present a public health challenge that permeates the individual provider, patient, and health systems levels [21].

Stress among maternal healthcare providers, a group at significant risk due to the nature of care provision in maternal and child health settings, has been associated with disrespect and abuse of women during childbirth [22, 23]. Yet, the empirical evidence on stress and burnout among maternity providers in sub-Saharan Africa (SSA) is scarce. A systematic review [11] of burnout among maternity providers (midwives) in Uganda [24] and Senegal documented high levels of burnout among midwives [25]. Further, two recent studies in Kenya also found high levels of burnout (88.6%) among nurses in maternity wards [26], while the second study, published by our team, reported high stress (96.5%) and burnout (84.6%) among maternity providers [22]. The study in Kenya, which was also the first study among providers in SSA to examine physiological measures of stress, also documented on average low heart rate variability (HRV) and high hair cortisol levels, indicative of accumulated stress [22].

Maternal healthcare providers in SSA are burdened by the universal challenge of working in highly stressful environments. Providers in this setting are stressed due to staff shortages [22, 27], poor working conditions including lack of water and poor sanitation, and frustrations due to the absence of medications and limited supplies and equipment [28, 29]. These challenges are further exacerbated by feelings of inadequacy in the face of increasing maternal and new-born mortality [29], financial strain from insufficient salaries and incentives, as well as perceived mistreatment by patients, colleagues, and superiors [23]. The mounting evidence for the pervasive and persistent levels of stress among maternal healthcare providers and the documented negative impacts on providers as well as patients, underscore the need for studies that examine healthcare providers’ experience of stress and burnout.

Individual and sociodemographic factors associated with stress and burnout among healthcare providers reported by previous studies include self-rated health status, experiencing disrespect from colleagues, overcommitment, marital status and educational status. For example, female healthcare providers [15], those with higher educational levels [30], those who are not married, those who experienced disrespect from colleague and healthcare providers who are overcommitted [23] were found to experience high levels of stress and burnout. However, Alfuqaha, et al. 2018 [31] found no significant association between gender, marital status, educational status and experience with burnout among nurses working in Jordan. These inconsistencies in the current literature suggest the need for further studies to understand the individual and sociodemographic factors associated with stress and burnout among healthcare providers.

We sought to build on our previous research in Kenya [22], to (1) assess levels of stress and burnout among maternity providers and support staff in Ghana, and (2) identify individual and situational factors associated with maternity provider stress, burnout, and stress-related physiologic measures. We hypothesized that perceived stress, burnout and physiological measures are influenced by individual level factors (including demographic and socioeconomic factors) as well as by situational factors related to job demands and resources [32]. We further hypothesized that perceived stress mediates the relationship between these potential individual and situational stressors and burnout and physiological responses. Our study provides critical data to inform strategies to address healthcare provider stress in various settings in SSA.

## Methods

### Study setting

This cross-sectional study was conducted across 15 administrative municipalities/districts in the Upper East region (UER) of Ghana. Each district has approximately 1 district hospital with the regional hospital serving as the referral facility. HCW-patient ratio is about four doctors and 200 nurses per 100,000 people (1:27,652 doctor-patient and 1:500 nurse-patient ratios) [33].

### Sampling and recruitment

Maternal healthcare providers, defined as nurses, midwives, doctors, and support staff working in maternity and maternity support, were purposively sampled from across 19 high delivery health facilities within the 15 districts in the region. A high delivery facility was defined as a facility with an average of 75 births per month in the base year—2019. All maternal healthcare providers in the selected facilities with more than 6 months of work experience were eligible to participate. We obtained the complete list of maternity providers from sampled health centers from the district directors of health services while that of the hospitals were obtained from either the hospital administrators or the medical officer in-charge of the facility. We spent 3 days in every facility inviting all providers on our list to participate in our study. In total we approached about 187 health workers, with 37 refusals, to achieve our target sample size of 150; a response rate of about 80%.

### Inclusivity in global research

Additional information regarding the ethical, cultural, and scientific considerations specific to inclusivity in global research is included in S1 Checklist.

### Data collection

Survey data were collected from October 2020 to January 2021, utilizing structured surveys among n = 150 maternity healthcare providers. Two trained research officers—1 male and 1 female–who are staff of the Navrongo Health Research Centre surveyed all the providers. Providers only participated in the interviews after providing written informed consent. All the interviews were conducted in English at private locations either at the homes of the providers or at the health facilities when providers were less busy or had closed from work. Each interview lasted about an hour.

### Outcome measures

Outcomes included psychological and physiological measures. Psychological measures included self-reported perceived stress and burnout while the physiological measures assessed the two primary stress response systems: parasympathetic nervous system assessed with provider’s resting heart rate variability (HRV) (i.e., vagal tone) and provider’s HPA axis response in the months preceding data collection (i.e., hair cortisol). These measures have been described in detail previously [22].

Perceived stress: we used the Cohen Perceived Stress Scale (PSS), a globally validated measure of perceived stress [34–36]. The ten-items in the PSS assess the degree of unpredictability, uncontrollability, and overload respondents experienced in the past month. The PSS has also undergone substantial testing for its validity and reliability, with internal consistency ranging from alphas of 0.69–0.91 across global studies [35–37]. The summative PSS score ranges from 0 to 40: 0–13 is considered low stress, 14–26 moderate stress, and 27–40 high perceived stress [34].

Burnout: we used the Shirom-Melamed Burnout Measure (SMBM), which assesses the degree of emotional, physical, and mental exhaustion caused by stress [38]. The SMBM has undergone psychometric testing in various populations with strong evidence for its validity and reliability in different populations including adult workers [39] and healthcare providers [40]. Reliability coefficients have exceeded 0.70 in most studies with adult workers in human service professions. Internal reliability testing with the sample in our study found a Cronbach alpha of 0.87. The 14-item SMBM has three subscales for physical fatigue (6 items), emotional exhaustion (3 items) and cognitive weariness (5 items). A mean burnout index was calculated for each participant, with scores ranging from 1 to 7, with scores of ≤2.0 considered as no burnout, 2.1–3.74 as moderate burnout and ≥ 3.75 as high burnout.

Heart rate variability (HRV): HRV was measured using the CorSense finger monitor by Elite HRV.41 The sensor was placed on the respondent’s index finger, with readings transmitted via blue tooth to the Elite HRV app on a phone. The reading was taken in the seated position for 5 mins in the middle of the interview after completing a series of questions on stress and burnout. HRV is a measure of the beat-to-beat interval between consecutive heart beats to estimate cardiac vagal tone [41, 42]. The time between R waves (R-R interval) in the QRS complex in electrocardiogram (ECG) of each participant was assessed using the Elite HRV software. The time domain measures including the RMSSD (root mean square of successive differences in RR intervals), lnRMSSD (the natural log of the RMSSD) and SDNN (standard deviation of all normal RR intervals) were used due to their utility and simplicity in short-term assessments. HRV measures were auto cleaned by the Elite HRV software to eliminate artifact [43]. LnRMSSD and SDNN scores were then calculated for each individual. Research has demonstrated the robust utility of cardiac vagal tone as a measure of stress [41]. These indices reflect parasympathetic nervous system (PNS) activity, with higher vagal tone associated with better physical and mental health [44]. Additionally, PNS activity is associated with relaxation and calm states. Broadly, higher levels of cardiac vagal tone are associated with younger age, better physical fitness and overall health, while low levels have been linked to depression, anxiety, high stress, burnout, mortality and morbidity [42, 45, 46].

Hair cortisol: we measured the downstream hormone secreted by the adrenal glands when the HPA axis is stimulated (typically measured in blood, saliva, urine, and hair) [47]. Hair cortisol levels, reflect cumulative secretion within that hair growth period [48, 49]. Each 1 cm of hair from the scalp assesses stress levels for the prior month, with high levels indicative of chronic or sustained stress [5, 50]. While there are no specified cut-offs for cortisol levels and stress, cortisol levels are higher in people with chronic stress and those with various health conditions [47]. One reference range reported for cortisol in hair is 17.7–153.2 pg/mg of hair (median 46.1 pg/mg) [51]. A more recent study reported hair cortisol concentration reference with low levels (40–128 pg/mg) and high (182–520 pg/mg) [52]. We obtained samples from only 90 respondents, due in part to cultural norms associated with donating/cutting hair and because some respondents did not have enough hair to provide a sample. Samples were processed at the Stress Physiology Investigative Team (SPIT) lab at Iowa State University (analytic processes are described elsewhere) [22].

Independent variables: We collected data on demographics (age, gender, marital status, parity), socioeconomic status (education, income, perceived social status and accomplishments), physical health (self-reported health status, chronic diseases, and exercise), health system indicators (facility type and level), as well as provider role and experience [22].

### Analysis

Data were analysed using Stata version 14.1 (College Station, TX). Initial analysis comprised of factor analysis of percieved stress and burnout related items to ascertain construct validity, and Cronbach’s alpha to asses internal consistency reliability. We performed these assessments for appropriateness for our sample prior to summative scores generation. Normality of the summative scores were tested using Shapiro-Wilk normality test and analysis conducted after necessary data transfomation to attain normality of the data.

We generated the descriptive statistics (means and proportions) to examine the distribution of explanatory and explained variables. At the bivariate level, we used crosstabulation, correlations, and unadjustered mixed-effect linear regression models with robust error, controlling for clustering at health facility. At the multivariate level, we included only variables with at least an overall p-value of less then 0.2 from bivariate analysis to avoid negative confounding. Factors associated with our outcomes of interest were obtained using mixed-effect linear regression models with robust errors, controlling for clustering at health facility. Model fit was determined using the Akaike Information Criterion. A structural equation model was applied to ascertain whether perceived stress mediated the effect of overcommittment on burnout.

### Ethics

Study approvals were provided by the Institutional Review Boards for Protection of Human Subjects of University of California, San Francisco [IRB 20–31248], and the Navrongo Health Research Centre Institutional Review Board (NHRCIRB386). We sought further permission from the Upper East Regional Director of Health Services, the District Directors of Health and the facility heads to conduct the study in their health facilities in the region. All participants provided written informed consent. The consent document detailed the study procedures, voluntary participation, risks and benefits of the study, compensation, privacy and confidentiality, the right to ask questions and contact information of the investigators and the ethics committees. A copy of the information sheet and the signed consent document were given to the participants for their reference.

## Results

### Demographics

Of the 150 providers who participated in the study, most (97.3%) were female. Most (71.3%) were above 29 years of age, with 56.0% aged 30 to 39 years, and 15.3% aged 40 to 52 years. Near all (96.7%) were nurses/midwives; few were ward aides/assistants (3.3%). Nearly two thirds (61.4%) worked in government hospitals, while 21.3% worked in government health centers, and 17.3% in Mission/private facilities. About half (46.0%) had been in their positions for five years or less. Due to missing data on some outcome measures, the analyzed sample varied: the sample was 150 for the perceived stress, 149 for burnout and HRV, and 90 for cortisol; the overall distribution of respondents was relatively similar in each of these analyzed samples (Table 1).

**Table 1:** Participant demographics and selected independent variables (N = 150).

Characteristic	Category	Perceived stress (N = 150)	Burnout (N = 149)	HRV (N = 149)	Hair cortisol (N = 90)
		No. (%)	No. (%)	No. (%)	No. (%)
Females		146 (97.3)	145 (97.3)	145 (97.3)	90 (100)
Age	23 to 29 years	43 (28.7)	43 (28.9)	42 (28.2)	27 (30.0)
	30 to 39 years	84 (56.0)	83 (55.7)	84 (56.4)	49 (54.4)
	40 to 52 years	23 (15.3)	23 (15.4)	23 (15.4)	14 (15.6)
Marital status	Married	111 (74.0)	110 (73.8)	110 (73.8)	69 (76.7)
	All single	39 (26.0)	39 (26.2)	39 (26.2)	21 (23.3)
Number of children	No children	35 (23.3)	35 (23.5)	35 (23.5)	14 (15.6)
	1 to 2 children	84 (56.0)	83 (55.7)	44 (29.5)	57 (63.3)
	3 or more children	31 (20.7)	31 (20.8)	70 (47.0)	19 (21.1)
Education level	College and below	128 (85.3)	127 (85.2)	127 (85.2)	77 (85.6)
	University and above	22 (14.7)	22 (14.8)	22 (14.8)	13 (14.4)
Monthly salary	Less than 2000 GHS (250 USD)	113 (75.3)	112 (75.2)	112 (75.2)	68 (75.6)
	2000–3000 GHS (250–375 USD)	37 (24.7)	37 (24.8)	37 (24.8)	22 (24.5)
Perceived social status of family growing up	Bottom half	104 (69.3)	103 (69.1)	103 (69.1)	59 (65.6)
	Upper half	46 (30.7)	46 (30.9)	46 (30.9)	31 (34.4)
Perceived social status of self	Bottom half	59 (39.3)	59 (39.6)	59 (39.6)	32 (35.6)
	Upper half	91 (60.7)	90 (60.4)	90 (60.4)	58 (64.4)
Perceived accomplishments in life	Less than you hoped	120 (80.0)	119 (79.9)	119 (79.9)	72 (80.0)
	Exactly/more than what you hoped	30 (20.0)	30 (20.1)	30 (20.1)	18 (20.0)
Self-rated health	Fair/poor	18 (12.0)	18 (12.1)	18 (12.1)	13 (14.4)
	Good/Very good/Excellent	132 (88.0)	131 (87.9)	131 (87.9)	77 (85.6)
Has chronic health condition		25 (16.7)	25 (16.8)	24 (16.1)	17 (18.9)
Frequency of exercise	Never/less than once a week	92 (61.3)	91 (61.1)	91 (61.1)	60 (66.7)
	Once or more per week	58 (38.7)	58 (38.9)	58 (38.9)	30 (33.3)
Facility type	Govt hospital	92 (61.4)	91 (61.1)	91 (61.1)	62 (68.9)
	Govt health center/Dispensary	32 (21.3)	32 (21.5)	32 (21.5)	19 (21.1)
	Mission/private	26 (17.3)	26 (17.4)	26 (17.5)	9 (10.0)
Position	Nurse/Midwife	145 (96.7)	144 (96.6)	144 (96.6)	144 (96.6)
	Support	5 (3.3)	5 (3.4)	5 (3.4)	5 (3.4)
Years as provider	0 to 5 years	69 (46.0)	68 (45.6)	68 (45.6)	41 (45.6)
	6 to 10 years	43 (28.7)	43 (28.9)	43 (28.9)	25 (27.8)
	More than 10 years	38 (25.3)	38 (25.5)	38 (25.5)	24 (26.7)
Workdays per Week	5 or fewer days	65 (43.3)	65 (43.6)	65 (43.6)	38 (42.2)
	More than 5 days	85 (56.7)	84 (56.4)	84 (56.4)	52 (57.8)
Work hours per day	8 or fewer hours	123 (82.0)	122 (81.9)	122 (81.9)	74 (82.2)
	More than 8 hours	27 (18.0)	27 (18.1)	27 (18.1)	16 (17.8)
Stressful interpersonal interactions	Experienced disrespect from superior in last year	60 (40.0)	60 (40.3)	60 (40.3)	36 (40.0)
	Experienced disrespect from colleague in last year	58 (38.9)	57 (38.5)	57 (38.5)	36 (40.0)
	Experienced disrespect from patient in last year	103 (69.1)	102 (68.9)	102 (68.9)	66 (74.2)
Overcommitment score: mean (SD)		149 17.2(2.32)	148 17.2(2.33)	148 17.2 (2.33)	89 17.6(1.19)

### Stress and burnout levels

On average, the PSS score was 1.47 (SD = 0.56) (Table 2), with over half (58.0%) of the participants scoring moderate/high stress (56.7% moderately stressed, and 1.3% highly stressed) based on the recommended cutoffs. The average burnout score was 2.45 (SD = 0.79), with 65.8% classified as having burnout (with 60.4% classified as having low burnout while 5.4% as high burnout). The average HRV score and cortisol levels were 56.07 (SD = 7.36) and 30.10 pg/mg (SD = 24.66), respectively. Perceived stress and burnout were moderately correlated (r = 0.50, p<0.001); however, HRV and hair cortisol levels were not significantly correlated with each other nor with perceived stress and burnout.

**Table 2:** Distribution of outcome measures (N = 150).

Continuous stress variables	N	Mean	SD	Median	Minimum	Maximum
Perceived stress score	150	1.47	0.56	1.4	0.1	3
Burnout score	149	2.45	0.79	2.36	1	4.93
Physical fatigue	149	3.08	1.06	3	1	6.17
Emotional exhaustion	149	1.59	0.90	1	1	4
Cognitive weariness	149	2.19	1.08	2.2	1	4.8
HRV measures						
HRV	149	56.07	7.36	56	38	72
Rmssd	149	42.90	21.07	38.65	11.72	109.54
lnRmssd	149	3.65	0.48	3.65	2.46	4.70
Sdnn	149	51.96	23.39	48.64	19.86	190.41
Hair cortisol level (pg/mg)	90	30.10	24.66	25.54	1.95	157.35
Log Cortisol	90	3.11	0.81	3.24	0.67	5.06
Categorical stress variables						
	Category		N	%		
Perceived stress from PSS	Low stress (0–13)		63	42.0		
	Moderate		85	56.7		
	High stress		2	1.3		
Burnout from SMBS	No burnout (< = 2.0)		51	34.7		
	Low burnout (2.1 to 3.75)		90	60.0		
	high burnout (> = 3.75)		8	5.3		

Notes:

HRV- Heart Rate Variability [53].

Rmssd- Root mean square of successive differences in RR intervals.

lnRmssd- Natural log of root mean square of successive differences in RR intervals.

Sdnn- standard deviation of NN intervals.

PSS- Cohen Perceived Stress Scale.

SMBS- Shirom-Melamed Burnout Measure.

### Individual and situational predictors

Most (56.7%) providers reported working more than 5 days a week, with many (82.0%) working 8 hours or less per day. Most (79.9%) felt they had ‘accomplished less than what they hoped in life’, while 18.8% ‘accomplished exactly what they had hoped’, and only 1.3% ‘accomplished more than what they had hoped’. Most (88.0%) providers rated themselves as in good/very good/excellent health. A little over a third experienced disrespect from superiors (40.0%) or colleagues (38.9%) in the last one year; more than two thirds (69.1%) experienced disrespect from patients in the last one year (Table 1).

### Bivariate analysis

Results from bivariate analyses indicate higher monthly salary as well as having experienced disrespect from superior, colleague, or patient in the last one year, and overcommitment were all significantly associated with higher perceived stress, while higher perceived social status of family growing up and having higher self-rated health (good/very good/excellent) were associated with lower perceived stress (S1 Table). Having 1–2 children, at least a university-level education, experiencing disrespect from patients in the last one year, and overcommitment, were significantly positively correlated with burnout. For physiological measures, none of the variables was significantly associated with HRV. Higher number of children and higher years of experience were significantly associated with lower cortisol levels while higher self-rated health, working for more than 8 hours, and having experienced disrespect from patients in last one year were significantly associated with higher cortisol levels (S1 Table).

### Multivariate analysis

#### Perceived stress

In multivariate analyses, significant factors associated with perceived stress included higher self-rated health, disrespect from patients in the last year, and overcommitment (Table 3). Providers who rated their health as ‘good/very good/excellent’ had lower perceived stress scores compared to those who rated their health as ‘fair/poor’ (β = -1.94, 95% CI: -3.50, -0.38). Those who had experienced disrespect from colleagues in the last year had higher perceived stress scores compared to those who had not experienced disrespect (β = 1.77, 95% CI: 0.50, 3.04); and each unit increase in overcommitment to work was associated with 0.62 higher perceived stress score (β = 0.62, 95% CI: 0.22, 1.02).

**Table 3:** Multilevel multivariate linear regression on outcome measures (N = 150).

Characteristic	Category	Perceived stress	Burnout	HRV (lnRMSSD)	Cortisol (log
		coefficient (95% CI)	coefficient (95% CI)	coefficient (95% CI)	coefficient (95% CI)
Age	23 to 29 years	-	-	Reference	Reference
	30 to 39 years	-	-	0.12 (-0.05,0.29)	0.30 (-0.06,0.65)
	40 to 52 years	-	-	-0.14 (-0.38,0.10)	0.48 (-0.06,1.03)
Marital status	Married	-	Reference	-	-
	All single	-	0.45 (0.08,0.82) *	-	-
Number of children	No children	-	Reference	-	Reference
	1 to 2 children	-	0.34 (-0.04,0.73)	-	-0.34 (-0.63, -0.04) *
	3 or more children	-	0.25 (-0.23,0.73)	-	-0.52 (-1.19,0.15)
Education level	College and below	-	Reference	-	-
	University and above	-	0.41 (0.03,0.79) *	-	-
Perceived social status of self	Bottom half	-	-	Reference	-
	Upper half	-	-	-0.14 (-0.29,0.02)	-
Self-rated health	Fair/poor	Reference	-	-	-
	Good/Very good/Excellent	-1.94 (-3.50, -0.38) *	-	-	-
Years as provider	0 to 5 years	-	-	-	Reference
	6 to 10 years	-	-	-	-0.21 (-0.51,0.10)
	More than 10 years	-	-	-	-0.58 (-1.16,0.01)
Workdays per Week	5 or fewer days	-	-	-	Reference
	More than 5 days	-	-	-	0.41 (0.02,0.80) *
Work hours per day	8 or fewer hours	-	-	Reference	-
	More than 8 hours	-	-	-0.12 (-0.32, 0.08)	-
Stressful interpersonal interactions	Experienced disrespect from superior in last year	-	-	-	-
	Experienced disrespect from colleague in last year	1.77 (0.50,3.04) **	-	-	-
	Experienced disrespect from patient in last year	1.67 (-0.60,3.94)	0.28 (-0.02,0.58)	-	0.52 (0.11,0.93) *
Overcommitment score: mean (SD)		0.62 (0.22,1.02) **	0.14 (0.07,0.19) ***	-	-
Random effects					
Health facility: identity var(_cons)		0.77 (0.02,28.33)	0.02 (0.001,0.41)	0.00	0.02 (0.001,0.29)
var (Residual)		25.65 (20.76,31.70)	0.67 (0.53,0.85)	0.21 (0.17,0.27)	0.49 (0.37,0.65)
ICC (Health facility)		0.03 (0.001,0.55)	0.03(0.002,0.39)	0.00	0.04 (0.002,0.38)
No. of groups		18	18	18	17
No. of observations		147	147	149	89

*<0.05,

**<0.01,

***<0. 001.

#### Burnout

Three variables were significantly associated with burnout at multivariable analysis (Table 3). Being single was associated with higher burnout scores as compared to being married (β = 0.45, 95% CI: 0.08, 0.82). Similarly, having at least a university education was significantly associated with higher burnout scores compared those with less than a university education (β = 0.41, 95% CI: 0.03, 0.79). Each unit increase in overcommitment to work was associated with 0.14 higher burnout scores.

#### Cardiac vagal tone

None of the variables were significantly associated with HRV in the multivariable analysis (Table 3).

#### Cortisol

The variables associated with cortisol levels at multivariate analysis included having parity, workdays per week, and experiencing disrespect from patients. Having 1 to 2 children was associated with lower cortisol levels compared not having any children (β = -0.34, 95% CI: -0.63, -0.04). Those who work for more than 5 days had higher cortisol levels, on average, compared to those who worked 5 or fewer days a week (β = 0.41, 95% CI: 0.02, 0.80). Similarly, those who had experienced disrespect from patient in last year had higher cortisol levels, on average, than those who had not (β = 0.52, 95% CI: 0.11, 0.93).

### Mediation analysis

Our results from the structural equation model are presented in Table 4. It shows that higher perceived stress was significantly associated with higher burnout scores (β = 0.07, 95% CI: 0.05, 0.10). Perceived stress accounted for 34.8% (p<0.001) of the effect of overcommitment on burnout, suggesting that perceived stress partially mediates the effect of overcommitment on burnout.

**Table 4:** Structural equation model for mediation of perceived stress on burnout (N = 147).

Characteristic	Burnout		
	Total effects	Direct effect	Indirect effects
	coefficient (95% CI)	coefficient (95% CI)	coefficient (95% CI)
Perceived stress	0.07 (0.05,0.10) ***	0.07 (0.05,0.10) ***	
Overcommitment score: mean (SD)	0.14 (0.08,0.20) ***	0.09 (0.03,0.15) **	0.05 (0.02,0.08) **

*p<0.05,

**p<0.01,

***p<0.001.

## Discussion

In this cross-sectional study of maternity health providers in the Upper East region of Ghana, we found that more than half of the providers reported moderate to high level of stress and 7 out of 10 had some level of burnout with 5.4% having high burnout that represent cause for clinical concern. Good or excellent self-rated health emerged as a protective factor to stress whilst excessive overcommitment to work and experiencing disrespect from colleagues in the last year were predictive for high perceived stress. Furthermore, being married and having college or lower degree emerged as protective factors for burnout whilst being single, having university degree or higher and overcommitment to work were predictive of high burnout. Perceived stress partially mediated the effect of overcommitment on burnout. Having 1 to 2 children, working for more than 5 days a week, and experiencing disrespect from patients in the last year were associated with higher cortisol levels.

The higher levels of perceived stress and burnout are consistent with prior studies among maternal healthcare providers [22, 25, 54, 55]. For example, Afulani and colleagues found that 96% of healthcare workers in maternity units in hospitals and health centers in Kenya were experiencing moderate to high stress, while 80% were experience some levels of burnout—with 20% experiencing clinical levels of burnout [22]. In another study during the COVID-19 pandemic, about two out three healthcare workers in Ghana surveyed were experiencing moderate to high stress and burnout [56]. A potential factor for these high rates of stress and burnout is the stressful work environments for healthcare providers, which has been recognized as a global crises [57]. Furthermore, maternity healthcare providers are constantly exposed to heavy workload which affects their physical performance as they try to harmonize professional standards and expectations from clients and their families. These providers are also exposed to various degree of trauma from complicated birth experiences and maternal and neonatal mortality, which can potentially increase their stress and burnout levels [58]. Due to the negative implications of high stress and burnout on mental and physical well-being and job performance [16, 17], our findings highlight the need for interventions to reduce stress among maternity providers.

A key finding from our study is that providers who experienced disrespect from colleagues and patients in the previous year were more likely to have higher levels of stress compared to those who did not experience any disrespect. This likely reflects the role of a toxic work environment, which is known to be a key source of workplace stress [59]. This is an important finding in this context of high stress and burnout, highlighting that, interventions to promote mutual respect among providers may help reduce workplace stress and burnout.

We also found that overcommitment predicted both higher perceived stress and burnout. Overcommitment to work, also referred to as ‘workaholism’ in some previous research [60], has been related to higher job stress and burnout and is associated with two particular dimensions of job burnout: emotional exhaustion and depersonalization [61]. Similarly, overcommitment is related to ‘drive’, which underlies as internal pressure to work and continuous thought about work [62]. People with high drive for work are more likely to stretch themselves to do more in hope of completing multiple tasks within a short period of time. Even though overcommitment represent a threat to employees’ well-being, it is an exaggerated form of job involvement, which is normally considered a desirable attitude by employers and supervisors because it induces employees to work harder and to maintain high levels of activity. However, this might push employees to burnout with subsequent decrease in productivity.

An interesting finding from our study is that having at least a university education was significantly associated with higher burnout compared to those with less than a university education. Our finding is contrary to the results of a recent study conducted in Jordan which found that lower educational level were associated with higher level of burnout among nurses [30], and another that found no association between burnout and education [31]. The inconsistent findings on the relationship between education and burnout is likely due to the complex ways in which education can influence burnout. For example, education may be protective by helping people to overcome difficulties or develop better coping mechanisms to reduce burnout. One the other hand findings such as ours may be due to an increased burden among more educated providers, such as the small number of people with university degrees in the selected health facilities being expected to take on more responsibilities, which leads to overcommitment and subsequent burnout. This implies supporting more maternity healthcare providers to obtain higher degrees could help reduce the workload on the few people with higher degrees and reverse the negative effects on burnout. Further studies are however needed to better understand this.

To our knowledge, only one study [22] in SSA has examined physiological measures of stress among maternity providers. As stated earlier, greater HRV is usually considered more adaptive since it reflects the ability of the ANS to dynamically adjust to environmental variations. We found that the HRV scores obtained in this study was lower than that obtained in the Kenya study. This implies that providers in Kenya may be having more challenges adapting physiologically to stressors. Similarly, the average cortisol level obtained in this study is lower than what we found among maternity providers in Kenya [22]. This difference in HRV and cortisol levels among the maternity providers in Ghana and Kenya support findings from previous studies that reported that providers in Kenya may be experiencing more stressful conditions compared to their counterparts in Ghana [27, 63]. This could be due to the differences in the provider-patient ratio in Kenya and Ghana [63]. We also found that having 1 to 2 children, working more than 5 days per week, and having experienced disrespect from patients in last one year predicted high level of cortisol among the study participants. These situations can heighten arousal for maternity providers thereby increasing their stress and burnout levels.

### Strengths and limitations

This study utilized psychosocial and physiological measures of stress and demonstrated the positive correlation of perceived stress and burnout, with stress as mediating factor for the effect of overcommitment on burnout. There was no significant correlation among other measures, concurrent with previous studies reporting inconsistent association between psychosocial and physiologic measures of stress [22, 64]. Additionally, we note the reduced probability of an association between HRV and cortisol, given our physiological measures assessed different stress response systems—a five-minute resting measure of HRV (cardiac vagal tone) and a retrospective HPA measure (cortisol). Further, HRV measures are limited in that they could be reflective of tonic or general state over previous months or could be reflective of an acute response to our research procedures. Finally, we note the limited generalizability of our findings, due to the relatively small sample size in one setting. The high rates of self-reported stress and burnout meant minimal variation in responses, possibly contributing to limited significant associations. Future studies should leverage larger sample sizes and varied settings to explore both the psychosocial and physiological stress measures to increase generalizability. Nonetheless, our findings underscore the high levels of stress among providers and the need for targeted interventions. To date, our novel study is the first to examine sources of stress among healthcare workers in Ghana, leveraging both psychosocial and physiological stress measures.

## Conclusion

We found high levels of stress and burnout among maternity providers in Ghana, underscoring the need for interventions to manage the effects of stress and burnout on maternity providers’ wellbeing, quality of care, and patient outcomes. Interventions should target underlying stressors as well as promote positive coping strategies to respond to both individual and work-related stressors. Healthcare management teams should assist providers in reducing their overcommitment, given its strong link to stress and burnout. Facilitating a respectful workplace culture could also help reduce stress and burnout. Future studies, including qualitative studies, should endeavor to understand the sources of stress across different provider populations, examine providers’ perceptions of the stressors, and identify coping mechanisms to mitigate the effects of stress and prevent burnout.

## Supporting information

S1 Checklist Inclusivity in global research. (DOCX)

S1 Table Multilevel bivariate linear regression on outcome measures. (DOCX)

## References

[1] Cheung S, Petersen S, McLellan T. Physiological strain and countermeasures with firefighting. *Scandinavian journal of medicine & science in sports*. 2010;20:103–116. doi: 10.1111/j.1600-0838.2010.01215.x 21029197

[2] Geenen R, Van Middendorp H, Bijlsma JW. The impact of stressors on health status and hypothalamic-pituitary-adrenal axis and autonomic nervous system responsiveness in rheumatoid arthritis. *Annals of the New York Academy of Sciences*. 2006;1069(1):77–97. doi: 10.1196/annals.1351.007 16855136

[3] Nicolaides NC, Kyratzi E, Lamprokostopoulou A, Chrousos GP, Charmandari E. Stress, the stress system and the role of glucocorticoids. *Neuroimmunomodulation*. 2015;22(1–2):6–19. doi: 10.1159/000362736 25227402

[4] Cuesta JM, Singer M. The stress response and critical illness: a review. *Critical care medicine*. 2012;40(12):3283–3289. doi: 10.1097/CCM.0b013e31826567eb 22975887

[5] McEwen BS. Stressed or stressed out: what is the difference? *Journal of Psychiatry and Neuroscience*. 2005;30(5):315–318. 16151535 PMC1197275

[6] Cox T, Kuk G, Leiter MP. Burnout, health, work stress, and organizational healthiness. In: *Professional burnout*. Routledge; 2017:177–193.

[7] Bridgeman PJ, Bridgeman MB, Barone J. Burnout syndrome among healthcare professionals. *The Bulletin of the American Society of Hospital Pharmacists*. 2018;75(3):147–152. doi: 10.2146/ajhp170460 29183877

[8] Yang Y, Hayes JA. Causes and consequences of burnout among mental health professionals: A practice-oriented review of recent empirical literature. *Psychotherapy*. 2020;57(3):426. doi: 10.1037/pst0000317 32463274

[9] Mata DA, Ramos MA, Bansal N, et al. Prevalence of depression and depressive symptoms among resident physicians: a systematic review and meta-analysis. *Jama*. 2015;314(22):2373–2383. doi: 10.1001/jama.2015.15845 26647259 PMC4866499

[10] Kuhn CM, Flanagan EM. Self-care as a professional imperative: physician burnout, depression, and suicide. *Canadian Journal of Anesthesia/Journal canadien d’anesthésie*. 2017;64(2):158–168. 10.1007/s12630-016-0781-0 27910035

[11] Dubale BW, Friedman LE, Chemali Z, et al. Systematic review of burnout among healthcare providers in sub-Saharan Africa. *BMC public health*. 2019;19(1):1–20. 31510975 10.1186/s12889-019-7566-7 PMC6737653

[12] Godoy LD, Rossignoli MT, Delfino-Pereira P, Garcia-Cairasco N, de Lima Umeoka EH. A comprehensive overview on stress neurobiology: basic concepts and clinical implications. *Frontiers in behavioral neuroscience*. 2018:127. doi: 10.3389/fnbeh.2018.00127 30034327 PMC6043787

[13] Chrousos GP. Stress and disorders of the stress system. *Nature reviews endocrinology*. 2009;5(7):374–381. doi: 10.1038/nrendo.2009.106 19488073

[14] Song PP, Walline JH. Physician burnout. *The Lancet*. 2020;395(10221):333. doi: 10.1016/S0140-6736(19)32480-8 32007159

[15] Mattei A, Fiasca F, Mazzei M, Abbossida V, Bianchini V. Burnout among healthcare workers at L’Aquila: its prevalence and associated factors. *Psychology*, *health & medicine*. 2017;22(10):1262–1270. doi: 10.1080/13548506.2017.1327667 28503931

[16] Goldberg DG, Soylu TG, Kitsantas P, Grady VM, Elward K, Nichols LM. Burnout among primary care providers and staff: evaluating the association with practice adaptive reserve and individual behaviors. *Journal of General Internal Medicine*. 2021;36(5):1222–1228. doi: 10.1007/s11606-020-06367-z 33420562 PMC8131495

[17] Bria M, Baban A, Dumitrascu DL. Systematic review of burnout risk factors among European healthcare professionals. *Cognition*, *Brain*, *Behavior*: *An Interdisciplinary Journal*. 2012;16(3):423–452.

[18] Maglalang DD, Sorensen G, Hopcia K, et al. Job and family demands and burnout among healthcare workers: The moderating role of workplace flexibility. *SSM-Population Health*. 2021;14:100802. doi: 10.1016/j.ssmph.2021.100802 33997249 PMC8102798

[19] Wallace JE, Lemaire JB, Ghali WA. Physician wellness: a missing quality indicator. *The lancet*. 2009;374(9702):1714–1721. doi: 10.1016/S0140-6736(09)61424-0 19914516

[20] Chemali Z, Ezzeddine F, Gelaye B, et al. Burnout among healthcare providers in the complex environment of the Middle East: a systematic review. *BMC public health*. 2019;19(1):1–21. 31640650 10.1186/s12889-019-7713-1 PMC6805482

[21] Lancet T. Physician burnout: a global crisis. In. Vol 3942019:93. 10.1016/S0140-6736(19)31573-9 31305255

[22] Afulani PA, Ongeri L, Kinyua J, Temmerman M, Mendes WB, Weiss SJ. Psychological and physiological stress and burnout among maternity providers in a rural county in Kenya: individual and situational predictors. *BMC public health*. 2021;21(1):1–16. 33676479 10.1186/s12889-021-10453-0 PMC7936594

[23] Afulani PA, Kelly AM, Buback L, Asunka J, Kirumbi L, Lyndon A. Providers’ perceptions of disrespect and abuse during childbirth: a mixed-methods study in Kenya. *Health policy and planning*. 2020;35(5):577–586. doi: 10.1093/heapol/czaa009 32154878 PMC7225569

[24] Muliira RS, Ssendikadiwa VB. Professional quality of life and associated factors among Ugandan midwives working in Mubende and Mityana rural districts. *Maternal and child health journal*. 2016;20(3):567–576. doi: 10.1007/s10995-015-1855-2 26525560

[25] Rouleau D, Fournier P, Philibert A, Mbengue B, Dumont A. The effects of midwives’ job satisfaction on burnout, intention to quit and turnover: a longitudinal study in Senegal. *Human resources for health*. 2012;10(1):1–14. doi: 10.1186/1478-4491-10-9 22546053 PMC3444355

[26] Muriithi J, Kariuki P. Work-related determinants of Nurses’ burnout in Pumwani Maternity Hospital, Nairobi City County, Kenya. *Asian Journal of Research in Nursing and Health*. 2020:36–49.

[27] Afulani PA, Aborigo RA, Nutor JJ, et al. Self-reported provision of person-centred maternity care among providers in Kenya and Ghana: scale validation and examination of associated factors. *BMJ global health*. 2021;6(12):e007415. doi: 10.1136/bmjgh-2021-007415 34853033 PMC8638154

[28] Buback L, Kinyua J, Akinyi B, Walker D, Afulani PA. Provider perceptions of lack of supportive care during childbirth: a mixed methods study in Kenya. *Health Care for Women International*. 2021:1–22. doi: 10.1080/07399332.2021.1961776 34534038 PMC9080303

[29] Merriel A, Larkin M, Hussein J, Makwenda C, Malata A, Coomarasamy A. Working lives of maternity healthcare workers in Malawi: an ethnography to identify ways to improve care. *AJOG Global Reports*. 2022;2(1):100032. doi: 10.1016/j.xagr.2021.100032 36274966 PMC9563393

[30] Alfuqaha OA, Alkawareek MY, Alsharah HS. Self-evaluation and professional status as predictors of burnout among nurses in Jordan. *PLoS One*. 2019;14(3):e0213935. doi: 10.1371/journal.pone.0213935 30901363 PMC6430417

[31] Alfuqaha O, Alsharah H. Burnout among Nurses and Teachers in Jordan: a comparative study. *Archives of Psychiatry and Psychotherapy*. 2018;20(2):55–65.

[32] Bakker AB, Demerouti E, Sanz-Vergel AI. Burnout and work engagement: The JD–R approach. *Annu Rev Organ Psychol Organ Behav*. 2014;1(1):389–411.

[33] Mo Health. *Holistic assessment of 2017 health sector programme of work*. 2018.

[34] Cohen S, Kamarck T, Mermelstein R. A global measure of perceived stress. *Journal of health and social behavior*. 1983:385–396. 6668417

[35] Garcia J, Hromi-Fiedler A, Mazur RE, et al. Persistent household food insecurity, HIV, and maternal stress in peri-urban Ghana. *BMC Public Health*. 2013;13(1):1–9. doi: 10.1186/1471-2458-13-215 23497026 PMC3608015

[36] Musana JW, Cohen CR, Kuppermann M, et al. Association of differential symptoms of stress to hair cortisol and cortisone concentrations among pregnant women in Kenya. *Stress*. 2020;23(5):556–566. doi: 10.1080/10253890.2019.1696305 31747807

[37] Lemma S, Gelaye B, Berhane Y, Worku A, Williams MA. Sleep quality and its psychological correlates among university students in Ethiopia: a cross-sectional study. *BMC Psychiatry*. 2012;12:237. doi: 10.1186/1471-244X-12-237 23270533 PMC3554495

[38] Shirom A, Melamed S. A comparison of the construct validity of two burnout measures in two groups of professionals. *International journal of stress management*. 2006;13(2):176.

[39] Schilling R, Colledge F, Brand S, Ludyga S, Gerber M. Psychometric properties and convergent validity of the Shirom–Melamed burnout measure in two German-speaking samples of adult workers and police officers. *Frontiers in psychiatry*. 2019;10:536. doi: 10.3389/fpsyt.2019.00536 31427997 PMC6688652

[40] Michel JS, Shifrin NV, Postier LE, Rotch MA, McGoey KM. A meta-analytic validation study of the Shirom–Melamed burnout measure: Examining variable relationships from a job demands–resources perspective. *Journal of Occupational Health Psychology*. 2022. doi: 10.1037/ocp0000334 35849372

[41] HRV E. What is Signal Quality? Signal Quality & Artifacts. 2022; https://help.elitehrv.com/article/351-what-is-signal-quality. Accessed March 7, 2022.

[42] Kim HG, Cheon EJ, Bai DS, Lee YH, Koo BH. Stress and Heart Rate Variability: A Meta-Analysis and Review of the Literature. *Psychiatry Investig*. 2018;15(3):235–245. doi: 10.30773/pi.2017.08.17 29486547 PMC5900369

[43] Järvelin-Pasanen S, Sinikallio S, Tarvainen MP. Heart rate variability and occupational stress-systematic review. *Ind Health*. 2018;56(6):500–511. doi: 10.2486/indhealth.2017-0190 29910218 PMC6258751

[44] Thayer JF, Åhs F, Fredrikson M, Sollers JJ III, Wager TD. A meta-analysis of heart rate variability and neuroimaging studies: implications for heart rate variability as a marker of stress and health. *Neuroscience & Biobehavioral Reviews*. 2012;36(2):747–756. doi: 10.1016/j.neubiorev.2011.11.009 22178086

[45] Lennartsson AK, Jonsdottir I, Sjörs A. Low heart rate variability in patients with clinical burnout. *Int J Psychophysiol*. 2016;110:171–178. doi: 10.1016/j.ijpsycho.2016.08.005 27535344

[46] Thayer JF, Lane RD. The role of vagal function in the risk for cardiovascular disease and mortality. *Biological psychology*. 2007;74(2):224–242. doi: 10.1016/j.biopsycho.2005.11.013 17182165

[47] Russell E, Koren G, Rieder M, Van Uum S. Hair cortisol as a biological marker of chronic stress: current status, future directions and unanswered questions. *Psychoneuroendocrinology*. 2012;37(5):589–601. doi: 10.1016/j.psyneuen.2011.09.009 21974976

[48] Kirschbaum C, Tietze A, Skoluda N, Dettenborn L. Hair as a retrospective calendar of cortisol production-Increased cortisol incorporation into hair in the third trimester of pregnancy. *Psychoneuroendocrinology*. 2009;34(1):32–37. doi: 10.1016/j.psyneuen.2008.08.024 18947933

[49] Pragst F, Balikova MA. State of the art in hair analysis for detection of drug and alcohol abuse. *Clin Chim Acta*. 2006;370(1–2):17–49. doi: 10.1016/j.cca.2006.02.019 16624267

[50] Maslach C, Schaufeli WB, Leiter MP. Job burnout. *Annual review of psychology*. 2001;52(1):397–422. doi: 10.1146/annurev.psych.52.1.397 11148311

[51] Sauvé B, Koren G, Walsh G, Tokmakejian S, Van Uum SH. Measurement of cortisol in human hair as a biomarker of systemic exposure. *Clin Invest Med*. 2007;30(5):E183–191. doi: 10.25011/cim.v30i5.2894 17892760

[52] Gonzalez D, Jacobsen D, Ibar C, et al. Hair Cortisol Measurement by an Automated Method. *Sci Rep*. 2019;9(1):8213. doi: 10.1038/s41598-019-44693-3 31160639 PMC6546685

[53] Shaffer F, Ginsberg JP. An Overview of Heart Rate Variability Metrics and Norms. *Front Public Health*. 2017;5:258. doi: 10.3389/fpubh.2017.00258 29034226 PMC5624990

[54] Banovcinova L, Baskova M. Sources of work-related stress and their effect on burnout in midwifery. *Procedia-Social and Behavioral Sciences*. 2014;132:248–254.

[55] Mohammad K, Al-Reda A, Aldalaykeh M, et al. Personal, professional and workplace factors associated with burnout in Jordanian midwives: a national study. *Midwifery*. 2020;89:102786. doi: 10.1016/j.midw.2020.102786 32619851

[56] Afulani PA, Gyamerah AO, Nutor JJ, et al. Inadequate preparedness for response to COVID-19 is associated with stress and burnout among healthcare workers in Ghana. *PloS one*. 2021;16(4):e0250294. doi: 10.1371/journal.pone.0250294 33861808 PMC8051822

[57] Bhatnagar G. Physician burnout. *Lancet*. 2020;395(10221):333. doi: 10.1016/S0140-6736(19)32612-1 32007160

[58] Sheen K, Spiby H, Slade P. Exposure to traumatic perinatal experiences and posttraumatic stress symptoms in midwives: prevalence and association with burnout. *International Journal of Nursing Studies*. 2015;52(2):578–587. doi: 10.1016/j.ijnurstu.2014.11.006 25561076

[59] Sheets OAF. Canadian Centre for Occupational Health and Safety Web site. In:2016.

[60] Vodanovich SJ, Piotrowski C, Wallace JC. The Relationship Between Workaholism and Health: A Report of Negative Findings. *Organization Development Journal*. 2007;25(1).

[61] Cheung F, Tang CS, Lim MSM, Koh JM. Workaholism on job burnout: A comparison between American and Chinese employees. *Frontiers in psychology*. 2018:2546. doi: 10.3389/fpsyg.2018.02546 30618967 PMC6298417

[62] Avanzi L, Perinelli E, Vignoli M, Junker NM, Balducci C. Unravelling work drive: a comparison between Workaholism and Overcommitment. *International Journal of Environmental Research and Public Health*. 2020;17(16):5755. doi: 10.3390/ijerph17165755 32784893 PMC7459690

[63] Afulani PA, Nutor JJ, Agbadi P, et al. Job satisfaction among healthcare workers in Ghana and Kenya during the COVID-19 pandemic: Role of perceived preparedness, stress, and burnout. *PLOS Global Public Health*. 2021;1(10):e0000022. 10.1371/journal.pgph.0000022 PMC10021773 36962085

[64] Staufenbiel SM, Penninx BW, Spijker AT, Elzinga BM, van Rossum EF. Hair cortisol, stress exposure, and mental health in humans: a systematic review. *Psychoneuroendocrinology*. 2013;38(8):1220–1235. doi: 10.1016/j.psyneuen.2012.11.015 23253896

