# Supplementary material for: Individual and situational predictors of psychological and physiological stress and burnout among maternity providers in Northern Ghana
Source: PLoS One. 2022 Dec 15;17(12):e0278457. doi: 10.1371/journal.pone.0278457 (PMC9754239; doi:10.1371/journal.pone.0278457)
Supplement: S1 Table — (DOCX) [file pone.0278457.s002.docx]

Table S1: Multilevel bivariate linear regression on outcome measures

| **Characteristic** | **Category** | **Perceived stress** | **Burnout** | **HRV (lnRMSSD)** | **Cortisol (log** |
| --- | --- | --- | --- | --- | --- |
|  |  | **coefficient (95% CI)** | **coefficient (95% CI)** | **coefficient (95% CI)** | **coefficient (95% CI)** |
| Gender | Female | Reference | Reference | Reference | Reference |
|  | Male | 0.90 (-5.25,7.06) | 0.17 (-0.72,1.07) | 0.20 (-0.35,0.75) | - |
| Age | 23 to 29 years | Reference | Reference | Reference | Reference |
|  | 30 to 39 years | 0.73 (-1.25,2.71) | 0.19 (-0.15,0.54) | 0.11 (-0.04,0.25) | -0.09 (-0.43,0.24) |
|  | 40 to 52 years | -1.19 (-4.48,2.10) | 0.17 (-0.30,0.64) | -0.17 (-0.35,0.02) | -0.32 (-0.76,0.12) |
| Marital status | Married | Reference | Reference | Reference | Reference |
|  | All single | 0.48 (-1.46,2.42) | 0.25 (-0.10,0.60) | -0.04 (-0.27,0.18) | 0.04 (-0.24,0.33) |
| Number of children | No children | Reference | Reference | Reference | Reference |
|  | 1 to 2 children | 0.57 (-1.43,2.57) | 0.27 (0.01,0.54) * | 0.06 (-0.12,0.24) | -0.29 (-0.58, -0.002) * |
|  | 3 or more children | 0.93 (-1.93,3.78) | 0.12 (-0.32,0.56) | -0.07 (-0.36,0.22) | -0.67 (-1.22, -0.12) ** |
| Education level | College and below | Reference | Reference | Reference | Reference |
|  | University and above | 0.38 (-1.75,2.51) | 0.55 (0.25,0.86) *** | 0.13 (-0.12,0.38) | -0.05 (-0.50,0.39) |
| Monthly salary | Below 2000 GHS | Reference | Reference | Reference | Reference |
|  | 2000-3000 GHS | 1.46 (0.18,2.74) * | 0.25 (-0.11,0.62) | -0.06 (-0.21,0.09) | -0.43 (-0.87,0.02) |
| Perceived social status of family growing up | Bottom half | Reference | Reference | Reference | Reference |
|  | Upper half | -1.40 (-2.47, -0.34) * | -0.02 (-0.28,0.25) | -0.11 (-0.26,0.04) | 0.06 (-0.22,0.35) |
| Perceived social status of self | Bottom half | Reference | Reference | Reference | Reference |
|  | Upper half | -0.50 (-2.29,1.29) | 0.05 (-0.26,0.36) | -0.12 (-0.30,0.05) | 0.08 (-0.16,0.33) |
| Perceived accomplishments in life | Less than you hoped | Reference | Reference | Reference | Reference |
|  | Exact/More than you hoped | -1.63 (-4.06,0.79) | -0.21 (-0.56,0.14) | 0.08 (-0.09,0.26) | 0.16 (-0.14,0.46) |
| Self-rated health | Fair/poor | Reference | Reference | Reference | Reference |
|  | Good/Very good/Excellent | -2.37 (-3.81, -0.93) ** | -0.28 (-0.73,0.17) | -0.06 (-0.34,0.22) | 0.37 (0.003,0.73) * |
| Has chronic health condition | | 0.94 (-0.32,2.19) | -0.003 (-0.44,0.43) | -0.03 (-0.28,0.22) | -0.22 (-0.65,0.21) |
| Frequency of exercise | Never/less than once a week | Reference | Reference | Reference | Reference |
|  | Once or more per week | -0.70 (-1.86,0.45) | -0.07 (-0.37,0.23) | 0.04 (-0.12,0.20) | 0.02 (-0.34,0.38) |
| Years as provider | 0 to 5 years | Reference | Reference | Reference | Reference |
|  | 6 to 10 years | 0.92 (-0.93,2.76) | -0.004 (-0.25,0.24) | 0.08 (-0.09,0.25) | -0.02 (-0.29,0.26) |
|  | More than 10 years | -1.16 (-3.47,1.14) | -0.02 (-0.34,0.29) | -0.03 (-0.19,0.12) | -0.47 (-0.92, -0.01) * |
| Workdays per Week | 5 or fewer days | Reference | Reference | Reference | Reference |
|  | More than 5 days | 0.43 (-0.84,1.70) | -0.03 (-0.32,0.25) | 0.02 (-0.12,0.15) | 0.36 (0.07,0.79) * |
| Work hours per day | 8 or fewer hours | Reference | Reference | Reference | Reference |
|  | More than 8 hours | 0.17 (-2.39,2.73) | -0.02 (-0.39,0.36) | -0.13 (-0.24,-0.02) | 0.02 (-0.52,0.56) |
| Stressful interpersonal interactions | Experienced disrespect from superior in last year | 1.52 (0.10,2.94) * | 0.10 (-0.10,0.31) | 0.01 (-0.13,0.16) | 0.20 (-0.06,0.47) |
|  | Experienced disrespect from colleague in last year | 2.48 (1.33,3.64) *** | 0.15 (-0.14,0.43) | -0.07 (-0.21,0.08) | 0.13 (-0.22,0.47) |
|  | Experienced disrespect from patient in last year | 2.71 (0.42,5.00) * | 0.43 (0.15,0.72) ** | -0.02 (-0.19,0.15) | 0.54 (0.12,0.97) * |
| Overcommitment score: mean (SD) | | 0.77 (0.40,1.14) *** | 0.15 (0.10,0.19) *** | -0.0001 (-0.03,0.03) | -0.03 (-0.08,0.03) |

*<0.05, **<005, ***<0.0001
